# Personal reflections on navigating plural values in the implementation of voluntary assisted dying in Victoria, Australia

**DOI:** 10.1007/s40592-024-00209-y

**Published:** 2024-10-23

**Authors:** Margaret O’Connor

**Affiliations:** Nursing and Midwifery, Faculty of Medicine, Nursing and Health Science, Monash University, McMahons Rd, Frankston, 3199 Australia

**Keywords:** VAD, Palliative care, Suffering

## Abstract

This paper is a personal reflection on involvement in the development of the first voluntary assisted dying legislation in Australia. Points of contention are discussed, where plural values were evident, as the legislation progressed towards implementation. Finally, ongoing areas of difficulty with the legislation are listed, where further thought is required to ensure ease of access for those in need.

## Introduction

Voluntary Assisted Dying (VAD) became legal in Victoria in 2019, the first Australian State to implement such legislation; at the time, it was said to be the most conservative assisted dying legislation in the world (White, Wilmott & Close 2019). With the subsequent passage of more liberal legislation across all Australian States and one Territory, Victoria’s legislation remains conservative.

This paper traces personal perspectives of the progress of the VAD legislation in the State of Victoria through its development into law, reflecting on issues that have occupied my thinking. I am the longest serving person in relation to this legislation, having been involved in the development of the Bill, the implementation of the Bill and am currently a member of the Review Board, which oversees each of the cases where a person has died by VAD.

Firstly, some personal framing, which has shaped my perspectives on this issue. I’ve spent almost 40 years at the bedsides of dying people, leading clinical palliative care teams, as well as researching in this area. The complexities of clinical and personal issues of active ending of life have become very stark over that time, thus neither black nor white, but very grey, a position long-held (O’Connor & Aranda 1996).

From my experience there seemed to be two kinds of people who make requests for assistance in dying– those people with a fixed and rational belief in their right to control their own end of life; and those who were worn down not just by their symptom burden and unbearable suffering, but also by the undignified process of dying; so much so that they seek relief through death.

In 2016 I attended a talk by Harvey Max Chochinov, an eminent Canadian psycho-oncologist (Frazee & Chochinov 2016), who was visiting Melbourne to discuss his leadership in implementing Medical Assistance in Dying (MAiD) in anticipation of the development of the Victorian legislation. In listening, I was very struck by his ability to discuss this issue without expressing a personal view. It was a ‘light-bulb moment’ that perhaps I could do that too.

## The process

The Victorian Government was about to select members of an advisory group, to assist the development of VAD legislation. Given what has been said above, about those groups of people who would be likely to seek assistance in dying, I was concerned that palliative care should not be forgotten in the process of forming the legislation. After all, palliative care can be of benefit to so many dying people including those seeking an active end to their life. I was accepted as part of a seven-person Ministerial Advisory Panel which drafted the legislation– chaired by a neurosurgeon. A health administrator, a consumer representative, a lawyer and three palliative care practitioners - two medical and I as a nurse, formed the Panel. It was a very diverse group, which ‘enabled discussion across medical, nursing, legal and ethical frameworks, to develop a professionally legitimate assisted dying process, which was adopted by the government in its entirety’ (Duckett 2019).

Over many months, the legislation developed, with the parliamentary legalistic process very evident, guided and directed by a government team of writers and policy experts. Consultation was extensive, with all manner of interest groups– more than 1000 individual representations and numerous group consultations around the State. (Duckett 2019; O’Connor et al. 2018). Many of the subsequently discovered practical difficulties of the legislation resulted from last-minute concessions resulting from negotiations that occurred during parliamentary debates.

What was of note in watching the Victorian parliamentary debates (and anecdotally in other States too), was the litany of harrowing, personal stories told by individual parliamentarians in arguing for VAD, often describing poor end-of-life care and inadequate palliative care. In listening to these stories, I worried as to how the legislation would be shaped by these experiences. Duckett’s careful analysis of these ‘pathographies’ (2020) indicates the use of ‘pathos’ (persuasion based on affecting the emotions of the audience); in that way confronting decision-makers with the lived reality of a bad death– converting statistics into someone’s loved one (Duckett 2020).

After the bill was enacted, there was a period of 18 months before it came into effect. (O’Connor et al. 2021) Perhaps naively I underestimated the immense task of respecting individual autonomy with decision-making over developing guidelines and policies - bringing on board healthcare and other institutions; working out the best way to deliver medication to applicants (a centralised statewide pharmacy); accommodating conscientious objectors & dealing with non-participating services; developing a database to maintain the applications and other administrative processes; and initiating a medical education program for providers. Implementation involved several working parties, comprising clinicians and health service managers to ensure the safe rollout of the process.

The VAD Review Board was established in 2018 to oversee the governance of the Act. Several functions are required under the Act, among them:– to review all cases; to promote compliance with requirements of the Act; to conduct research and promote continuous improvement; and, liaise with stakeholders like the Department of Health, the VAD navigators, the statewide pharmacy service and the Government Agency responsible for Births, Death & Marriages (Parliament of Victoria, 2017). The Board reports to Parliament every year and in its fifth year, the legislation required the Health Minister to conduct a review of the operations of the Act which is now underway.

Alongside my involvement in all this work, in 2018 I was privileged to receive a Winston Churchill Fellowship to study two specific aspects of VAD which I considered were not settled in Victoria - the relationship of palliative care services with people who request VAD; and the governance and reporting systems. Travel was undertaken, just as VAD became available to Victorians.

What was observed across Europe, Canada and the Unites States of America was systems and processes that were obviously ahead of Victoria in terms of development– most services had settled into a routine to determine who were providers and who were not, to accommodate a plurality of views. It was very consoling that the anticipatory anxiety and upset that I had left behind in Victoria, was probably time-limited. In Canada, I also learned of the importance of collecting early consistent and accurate data, when endeavouring to establish a national view of Medical Assistance in Dying (MAiD), because of the difficulties of aligning differing datasets from each Province. The importance of this visibility is that it provides accountability to the parliament and the general public. I have worked over the last few of years to proactively establish an Australian national minimum dataset.

So, after eight years of involvement in these varied roles, what are some of my reflections on the plural values that need to be accommodated in end-of-life care?

### Sanctity of life

Sanctity of life has been expounded down the centuries by numerous great thinkers (Clarke, 2023); it is a value, deeply embedded in Judeo-Christian societies, common in all Abrahamic faiths (Islam, Judaism and Christianity) as well as other religious traditions. A core tenet holds the intrinsic value of human life (sacred); therefore, human life has inherent worth and dignity at all stages of life, and therefore needs to be protected (O’Connor 2020). Dominant Christian and Jewish origins of sanctity of life are based in scripture in the book of Genesis when God created human beings in their own image. In the Qur’an, a story similar to that of Cain & Abel, where one brother murders another, and is an admonition against murder, supporting the value of life.^1^ In Buddhism and other faiths, there is an ethical principle of not causing harm to other living beings. The concept of Karma, commonly held by Hindus and Buddhists, offers an opportunity for personal growth arising out of suffering (Thrane 2010).

But– my struggle/discomfort is about holding an objective and theoretical valuing of life in this way, when an individual may not share that value, when the burden of end-of-life suffering overrides their own sense of the value of their remaining life.

### The nature of suffering

In major religious traditions suffering has been (is) justified by a prevalent religious view as a preparation for the next life, provides spiritual identification with a suffering god, and/or is a cleansing or reparation for one’s failings in life. Caitlin Maher in her book ’The Good Death’ (2023), considers this framing of dying to be of a time when there was nothing further that could be medically done for a dying person, so it was justified in altruistic religious terms. In Victorian England a ‘good death’ was marked by the embrace of suffering, rather than its elimination.

In contrast, in contemporary societies, suffering, appears to be increasingly less acceptable in general, but particularly for individuals facing the end of their life. It is universally the main underlying impetus for legislative change in relation to active assistance in dying (O’Connor et al. 2018). This may reflect increasing personal and community expectations for an individual to control what occurs at the end of their life, especially for ‘baby boomers’ for whom control seems essential. Although perhaps inevitable, suffering at the end of life and the deep personal anguish it may cause, remains an aspect of human life requiring further understanding and research.

At the end of life, suffering may pose a threat to one’s sense of self, especially when accompanied by the loss of individual autonomy, often expressed as a loss of control or dignity (Hall and Hill 2019). I’ve participated in too many conversations where a dying person has been searching for a source of their suffering - blaming themselves or others’ life choices, or reasoning that one’s actions are the cause. And my enduring question is: ‘what is the value of suffering when it is unwanted or imposed?’ There may be value only if suffering is chosen through acceptance, but making meaning of suffering remains a very personal choice.

Unbearable suffering is a key eligibility criterion for VAD. Despite many efforts like that of the eminent physician, Eric Cassell, (1994) an overall and agreed definition remains difficult and is problematic to measure. Suffering may be caused by physical pain, but is necessarily a whole-person experience, encompassing emotional, spiritual and social aspects of the person; thus, addressing and managing suffering is very complex. Cassell’s seminal work highlighted that the study of suffering is not usually included in medical curricula; thus, there is limited understanding amongst the medical profession as to the impact of suffering on the whole person. Perhaps relief of suffering at the end of life is a final frontier in controlling all aspects of human life?

### Conscientious objection

Conscientious objection is one area which I watch carefully, in respect of a clinician’s autonomous choice not to be involved in VAD. Under the Act, organisations can refuse to provide some services, appealing to particular values or principles.

While it may be expedient for an individual to verbalise their conscientious objection to VAD, there is more nuance required in thinking through autonomy. In the Victorian Act, there are six aspects of the legislation to which an individual might object, from providing information, to performing an active role (Parliament of Victoria, 2017). So, this may require time, in explaining to health professionals when teaching or talking about VAD, because more thought is required than a blanket refusal. For example, an individual may feel comfortable discussing VAD, but not comfortable playing an active role in the process.

Under the Act, coercion to take the medication is an offence, but it is not an offence to coerce a person *not* to apply for VAD or impede their access, because of clinician conscientious objection. Canadian Physician Sandy Buckman, wrote about becoming a ‘conscientious provider’, by making a considered and principled choice to becoming a provider (Buckman 2019).

### Palliative care services

In Victoria, like most places where VAD has been legalised, palliative care services benefitted from additional funding; rightly so, as palliative care can be of benefit to many dying people, whereas death using VAD comprises a very small percentage of all Victorian deaths (VAD Review Board 2023). But palliative care services have exhibited varying responses towards their involvement in VAD.

The internationally accepted definition of palliative care holds to a tenet that palliative care will not hasten or prolong death (Radbruch et al. 2016) and thus VAD has been viewed as incompatible with VAD. The service where I have most recently worked provides home-based palliative care through Melbourne’s northern suburbs. An early decision was made to not be a *provider* of VAD, but to continue care for an individual as they move through the VAD assessment process and not to abandon those who need care. When a request is made, this service works to link people with appropriate services - the Care Navigators mainly, who identify providers and assist with the process among other activities. While clinicians are respected and supported in their own view about being involved/not with someone seeking VAD, at no point is care discontinued. And my observation is that while it has taken time for some clinicians to become accustomed to a planned time of dying, most can now see that it is possible to continue excellent palliative care as the person prepares for their death.

I’m not pretending that all palliative care services have had such a smooth path, but a number work in this way, indicating to others what is possible. It was notable that at the national palliative care conference in 2023, there was a session on VAD, perhaps a sign of a growing recognition that VAD & palliative care are both key areas of concern at the end of life.

While there is literature that holds that palliative care cannot relieve all suffering (Cairns 2019), there is some argument that active assistance in dying would not be necessary if palliative care were more available/had more funding. In my experience, when a person requests assistance in dying, it has little to do with either the availability or quality of palliative care and perhaps they want both palliative care *and* VAD. This is borne out in the 2023 Review Board Report to the Victorian Parliament where 81% of those applying for VAD had also received palliative care involvement (VAD Review Board 2023). And I argue (often with the palliative care nay-sayers) that palliative care staff are experts in end-of-life care– if they won’t/can’t accompany people in their final days, then who will?

### Faith-influenced services

Despite the bad press about what faith-influenced services *don’t* do, most will work from an overriding concern for the person who is suffering, and temper responses by putting the person at the centre of their care. While not overtly assisting an individual, many will not obstruct their decision to choose VAD. Over the five years of the Victorian legislation, it has been observed that it is not necessarily the faith-influenced services where obstruction has been an issue. It is important to recognise the power of some clinicians in leadership roles who impose their personal conscientious objection stance on staff in particular clinical settings.

### Reasons for a request for VAD

It is interesting then that the major reasons for requesting assistance in dying around the world, are not unrelieved pain or other symptoms, but fear of being a burden and a loss of dignity (Downar et al. 2020).^2^

The fear of being a burden may reflect where we put our healthcare dollars. There is evident spending on medication and physical supports - easily measured interventions - but do dying people get access to the range of multi-disciplinary supports that might make life more comfortable and assist their independence for a little longer?- a bed in the home that can be altered into a sitting position; bathroom equipment so they can still shower unaided; regular in-home respite to give everyone a break; psychological and spiritual support to understand their emotional and mental issues; and support for carers who manage the homecare of their family member for the approximately 22 h a day when the health professionals are not present. There is a long way to go in truly practicing holistic healthcare.

## Some enduring issues in relation to the plurality of values that may be evident in relation to VAD

- **equity of access** issues remain the challenge in Victoria, mainly because of the small number of medical practitioner providers and of medical specialties, challenging the value of equity. While this may be reflective of the early days of implementation it does require careful monitoring in ensuring access across urban and rural settings. Unlike some other States, the only permitted role in Victorian legislation is for medical practitioners; other States where Nurse Practitioners are permitted to be providers, may lead the way for this to occur in Victoria.
- the ban on using **Telehealth** also challenges equity of access and denies person-centred care when desperately ill people need to travel to complete their assessments. It is an issue around the country, and it is hoped that the Federal Government will remove the Commonwealth Criminal Code’s prohibition of the use of a ‘carriage service’ like telehealth to conduct VAD activities.
- perhaps because of the area of Melbourne where I have most recently worked, I’m very aware of the difficulties of ‘**advertising’** the availability of VAD especially in different cultural groups, again challenging equity and the principle of advocacy for those in need. In the last Victorian Parliamentary report, 68% of applicants were born in Australia (VAD Review Board 2023). And while remembering that VAD is a very new procedure, generally, I’m unsure of the general community’s awareness and how that can be overcome - a sign in the tram-stop or a billboard on the freeway may not be socially acceptable! Together with the prohibition on health professionals being unable to raise VAD in end-of-life conversations, awareness-raising continues to be a significant issue. Perhaps ultimately, remembering our cultural aversion of death & dying, it remains one of those areas that is unknown by individuals until it is required.
- Sometimes the Board gets feedback about the **burdensome nature of the VAD process**. And this is a dilemma, how to respect person-centred care, and an individual’s own agency, in endeavouring to balance the enduring nature of the request and seriousness of the process, without making it overly burdensome for an individual who is suffering intolerably. There is sometimes a community expectation that VAD can occur quickly and while there is allowance in the Act for expedited requests, in no way should VAD be viewed as an emergency procedure.
- and there is an unusual dynamic I’ve noticed, as other States have developed their own legislation, with each State learning from Victoria and others. Victoria has gone from having ground-breaking legislation to **being the laggards**– for example, other States permit the raising of VAD in discussing end-of life options with individuals; other States permit nurses to have a role.

These issues and many others will be topics in the **Review of the Operations** of the Act. The Minister has limited the Review to the operations only, and despite close community interest, there is no political appetite for revision of the Act itself. In many ways this lack of political appetite limits what can be addressed, despite many operational aspects of the VAD process requiring attention.

## Conclusion

Active points of plurality have been illustrated in this paper, in describing a personal journey through the development of VAD in Victoria. Those who hold the seeming luxury of a firm stance on such a contentious topic are perhaps to be envied. But in engaging with legal representatives, health department personnel and policymakers through this process, and holding a decidedly clinical perspective on end-of-life care, my stance remains well and truly grey!

## Footnotes

1 See Qur’an 5:27–32.

2 A personal view is that there seems to be little excuse for people having unrelieved pain in Australia because of the vast choice of a range of medications and complementary therapies. While acknowledging this is a complex area of care, I’ve often (privately) wondered if it reflects the lack of skill of the clinician.
